# Supplementary material for: 3′-Sialyllactose alleviates bone loss by regulating bone homeostasis
Source: Commun Biol. 2024 Jan 19;7:110. doi: 10.1038/s42003-024-05796-4 (PMC10798968; doi:10.1038/s42003-024-05796-4)
Supplement: Supplementary file 2 — Supplementary information [file 42003_2024_5796_MOESM2_ESM.pdf]

SUPPLEMENTARY INFORMATION

CONTENT:

Supplementary Figures with figure legends: Supplementary Figure S1-3

Supplementary Tables: Supplementary Table 1-2

Supplementary Information

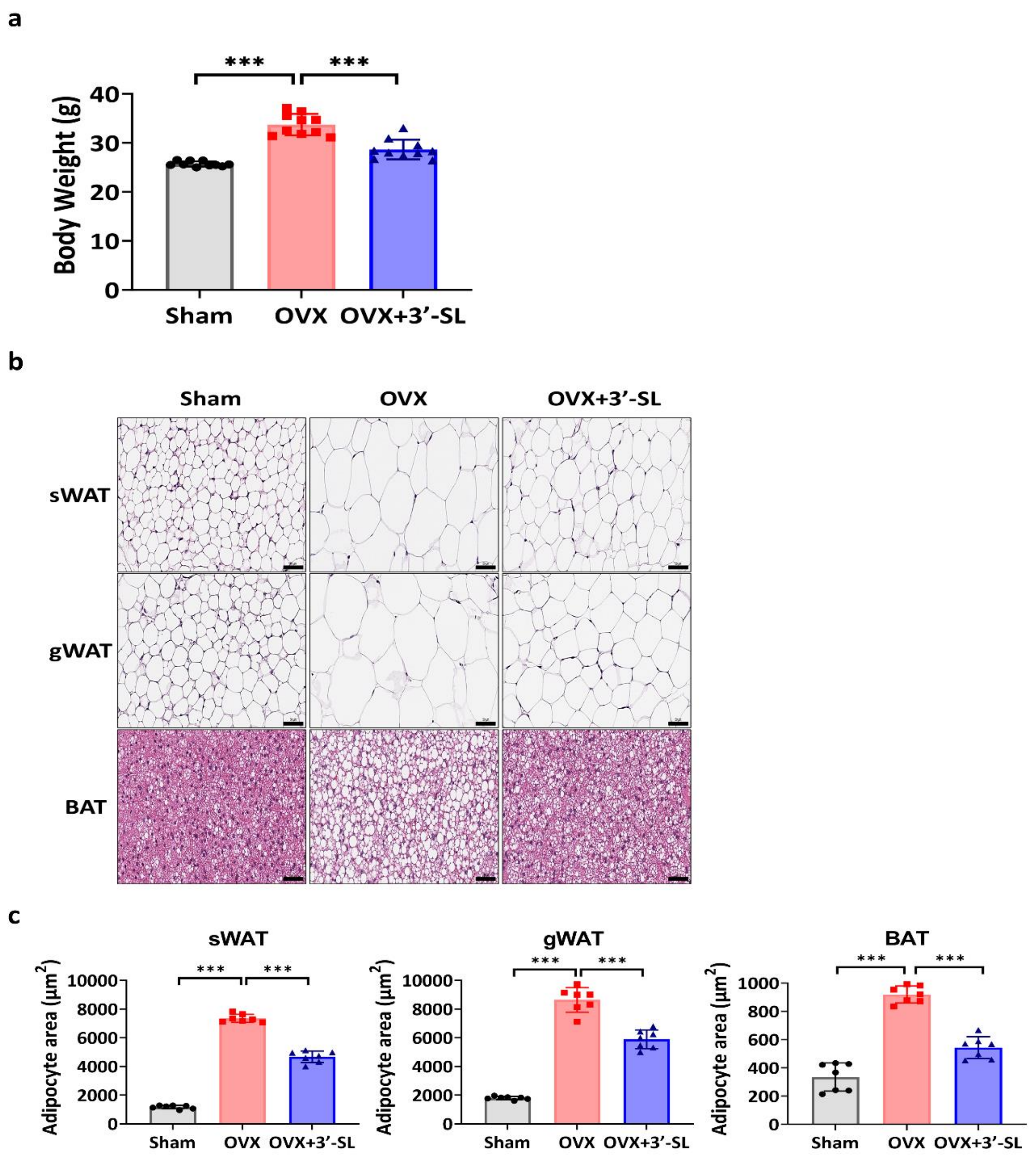

Supplementary Figure 1. 3′-sialyllactose reduces adipose tissue deposition and sizes of adipocytes. **a** Body weight comparison of 3′-sialyllactose (3′-SL) administered OVX-induced mice, OVX-induced mice, and Sham control group at termination of the study (*n* = 10 for each group). **b** Hematoxylin-eosin staining (H&E) of subcutaneous white adipose tissue (sWAT, top), gonadal white adipose tissue (gWAT, middle), and brown adipose tissue (BAT, bottom). Scale bar, 50 μm. **c** Quantification of adipocyte areas of sWAT, gWAT, and BAT using osteomeasure software (*n* = 7 for each group). Results are shown as means ± SDs. ****P* <0.001. *P*-values were determined using a one-way analysis of variance in **a** and **c**.

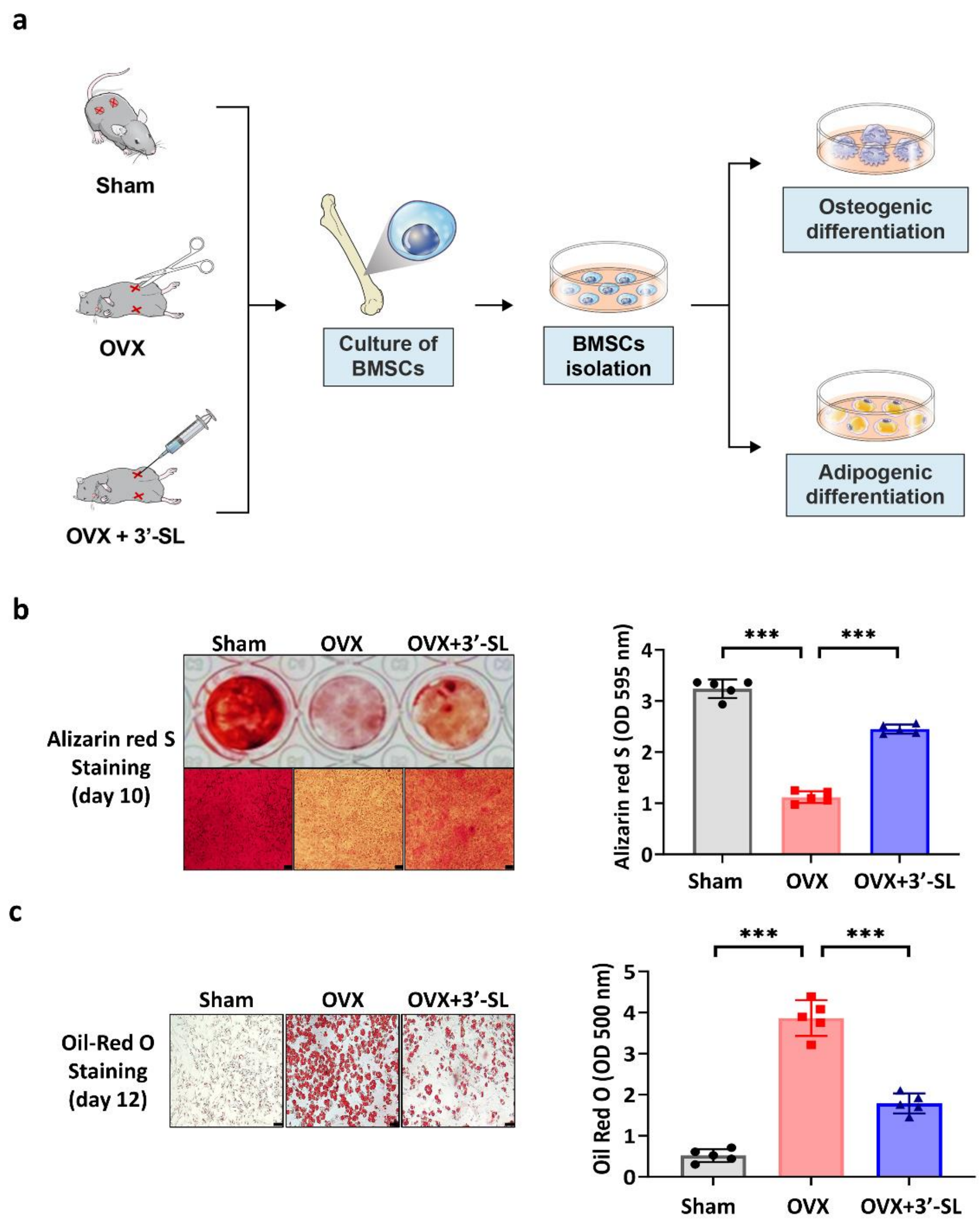

Supplementary Figure 2. Injection of 3′-sialyllactose promotes osteogenic differentiation and inhibits adipogenic differentiation of bone marrow stromal cells in OVX-induced mice. a The bone marrow stromal cells (BMSCs) were isolated from Sham control and OVX-induced with or without 3′-sialyllactose (3′-SL). The impacts of 3′-SL on osteogenic differentiation and adipogenic differentiation of BMSCs are shown. **b** The results of Alizarin red staining of the osteogenic BMSCs at day 10, and quantification of staining. Scale bar, 25 μm. **c** The results of Oil Red O staining of the adipogenic BMSCs at day 12, and quantification of staining. Scale bar, 50 μm. Results are shown as means ± SDs. *n* = 5 for each group. ****P* <0.001. *P*-values were determined using a one-way analysis of variance in **b** and **c**.

a

Figure 1 d

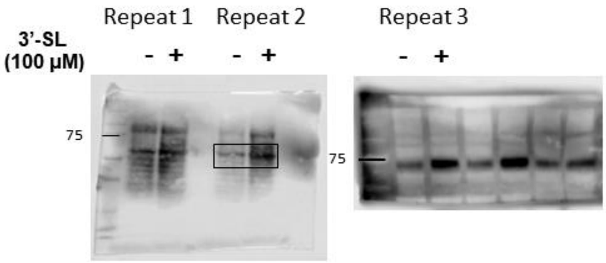

IB:RUNX2

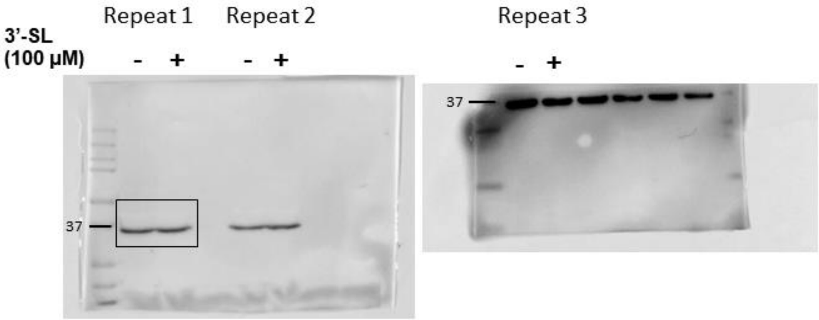

IB:GAPDH

Figure 1 d

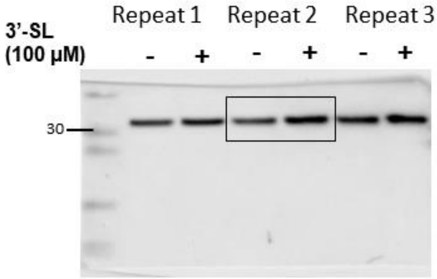

IB:IBSP

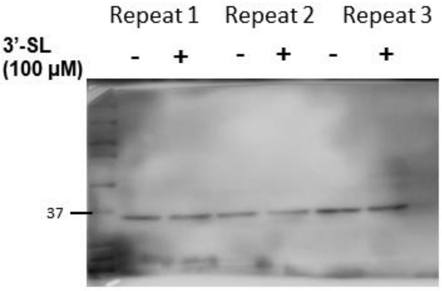

IB:GAPDH

Figure 1 d

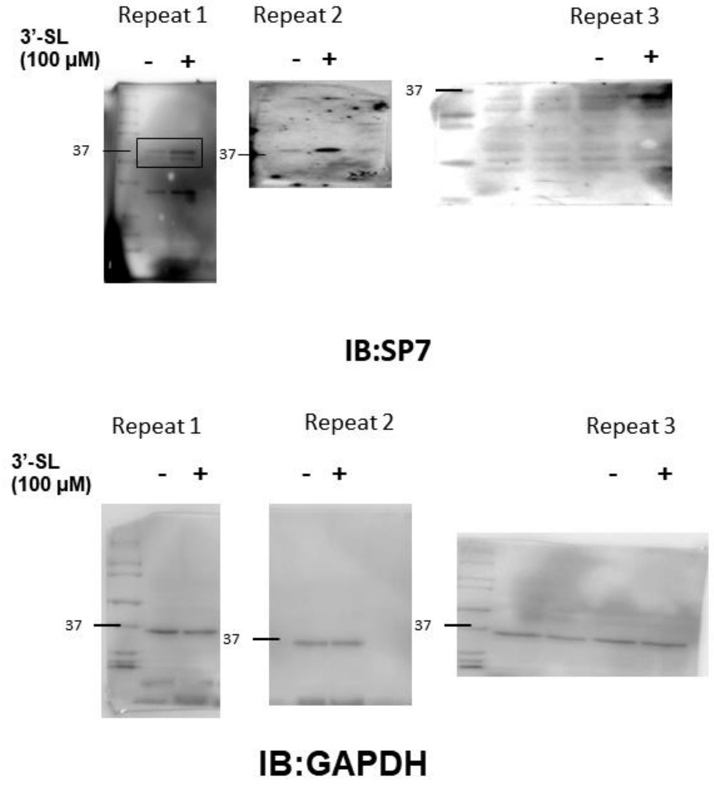

Figure 1 d

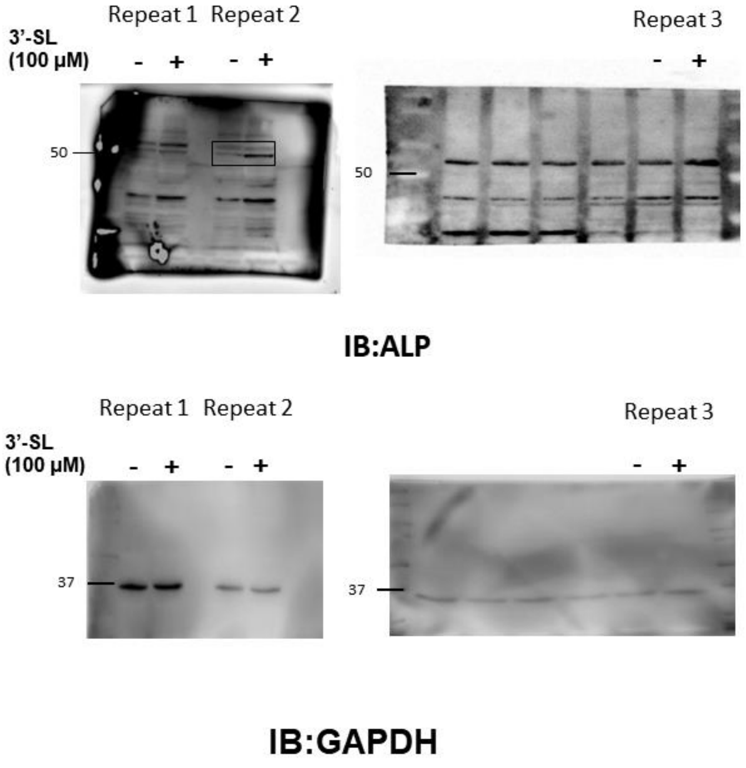

b

Figure 2 f

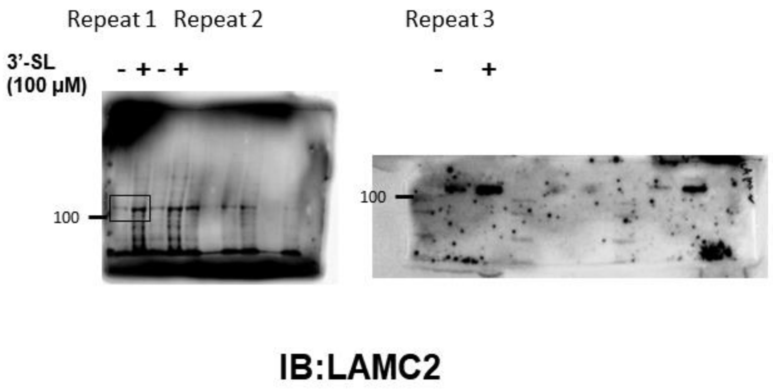

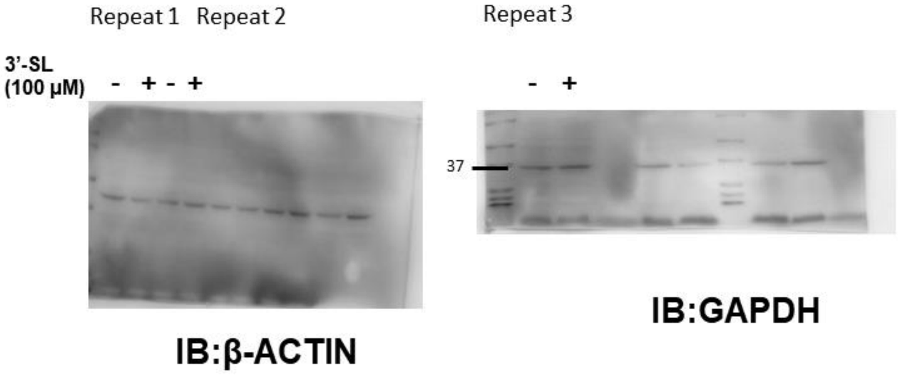

Figure 2 f

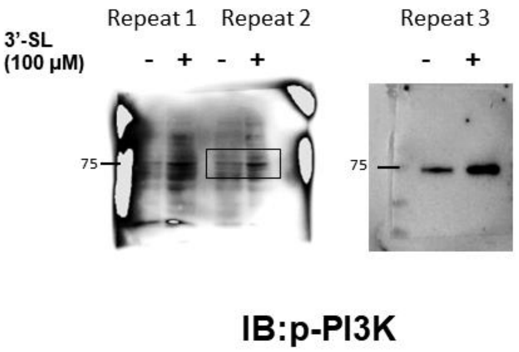

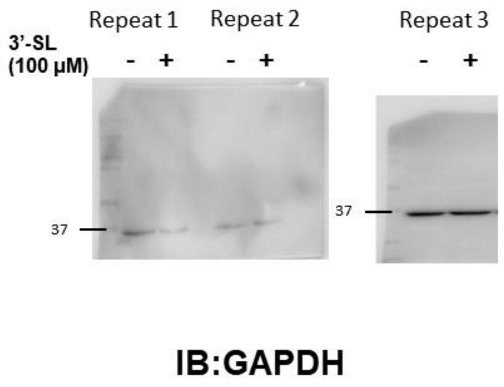

Figure 2 f

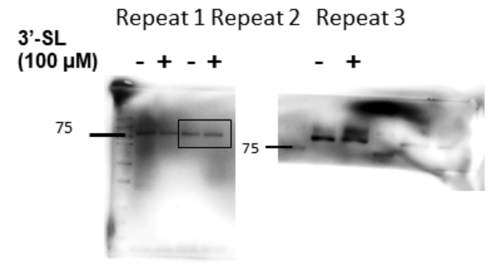

IB:PI3K

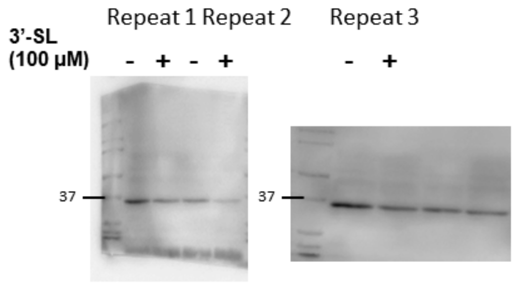

IB:GAPDH

Figure 2 f

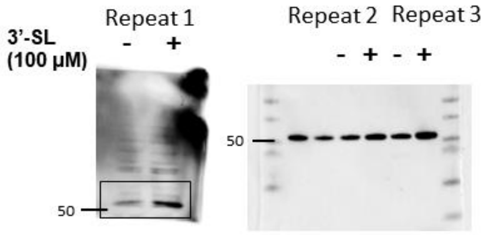

IB:p-AKT

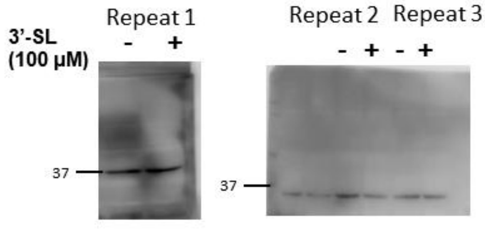

IB:GAPDH

Figure 2 f

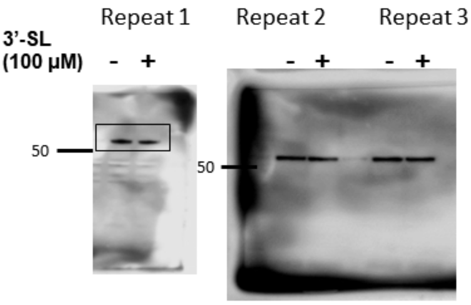

IB:AKT

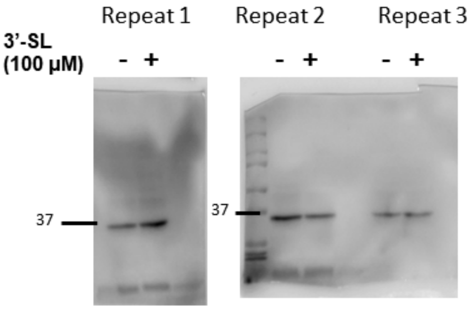

IB:GAPDH

Figure 2 f

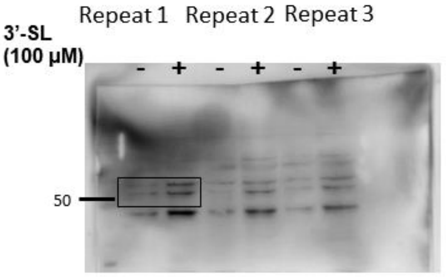

IB:ALP

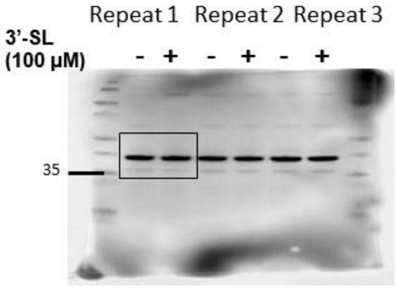

IB:GAPDH

Figure 2 f

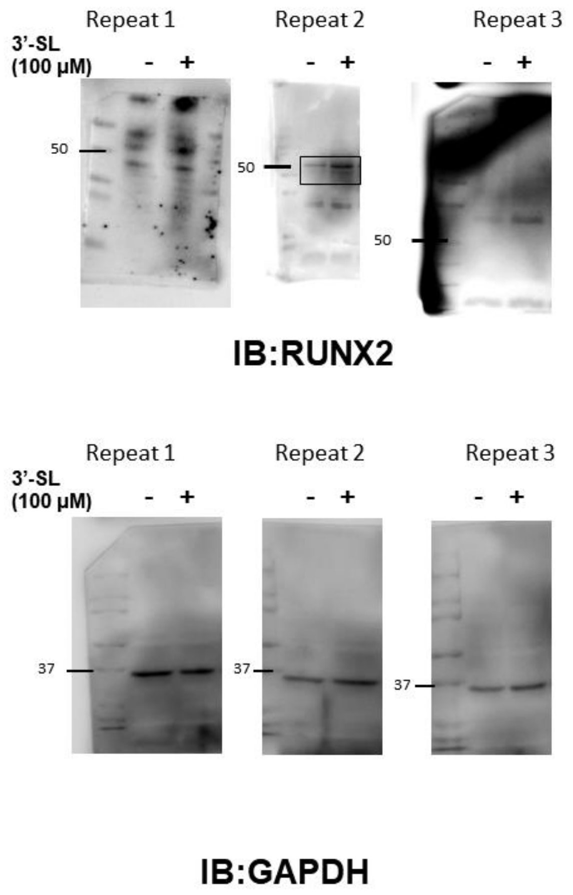

Figure 2 h

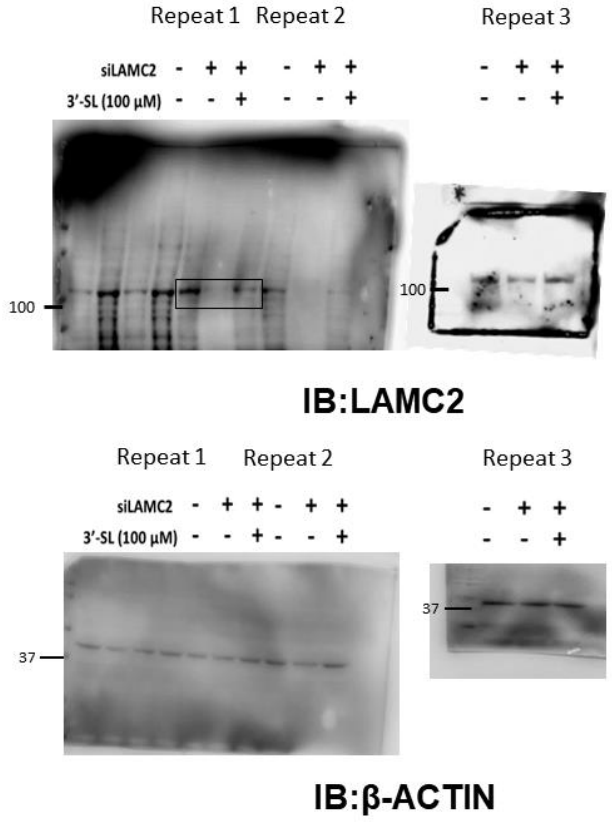

Figure 2 h

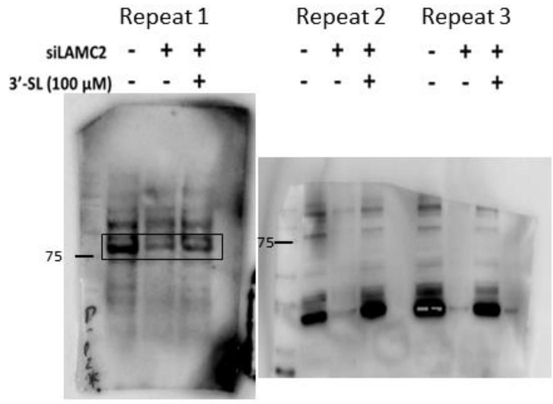

IB:p-PI3K

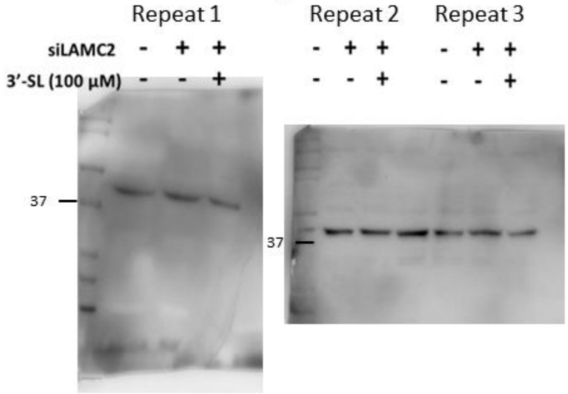

IB:β-ACTIN

Figure 2 h

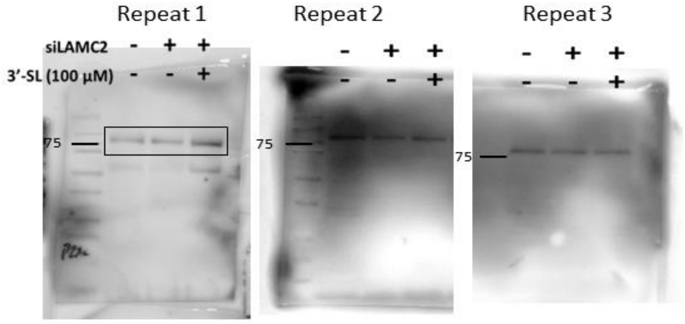

IB:PI3K

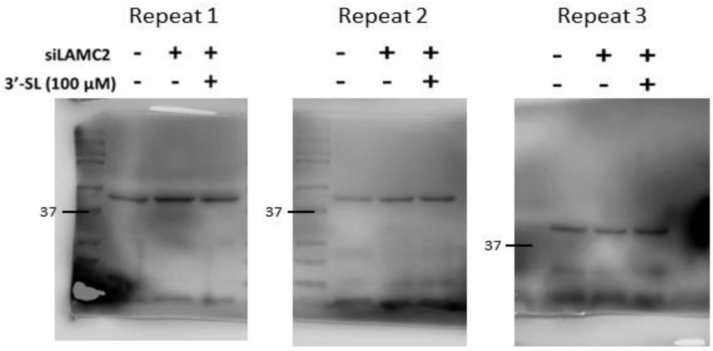

IB:β-ACTIN

Figure 2 h

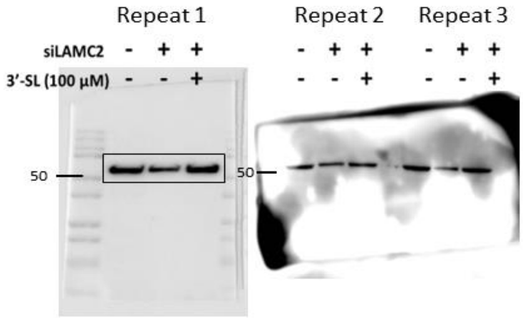

IB:p-AKT

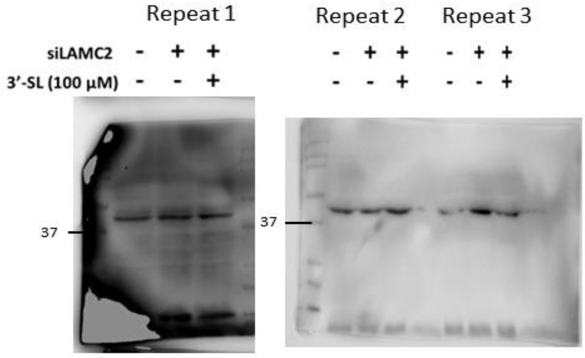

IB:β-ACTIN

Figure 2 h

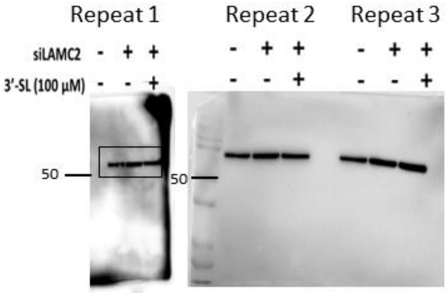

IB:AKT

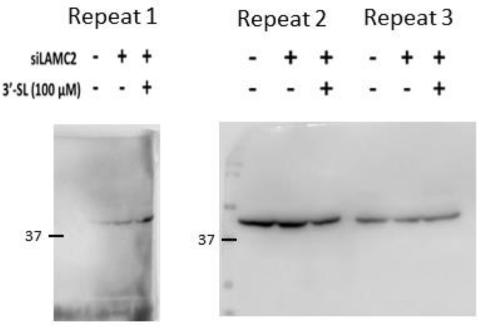

IB:β-ACTIN

Figure 2 h

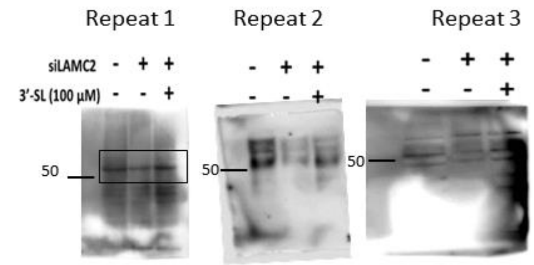

IB:RUNX2

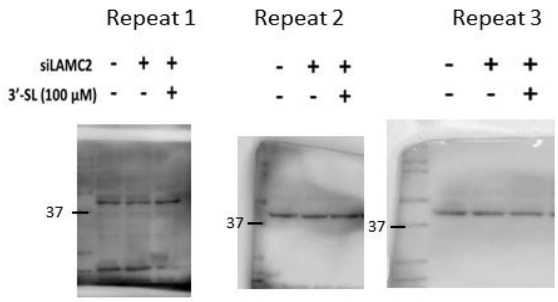

IB:β-ACTIN

Figure 2 h

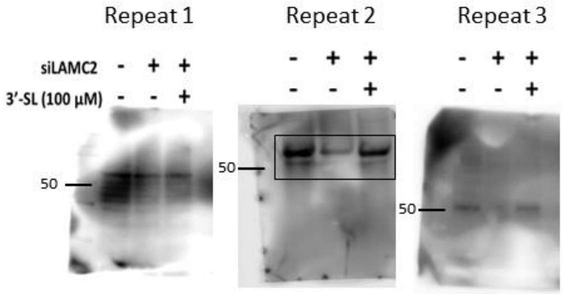

IB:ALP

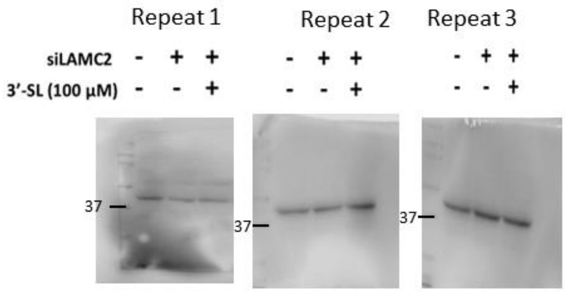

IB:β-ACTIN

Figure 2 h

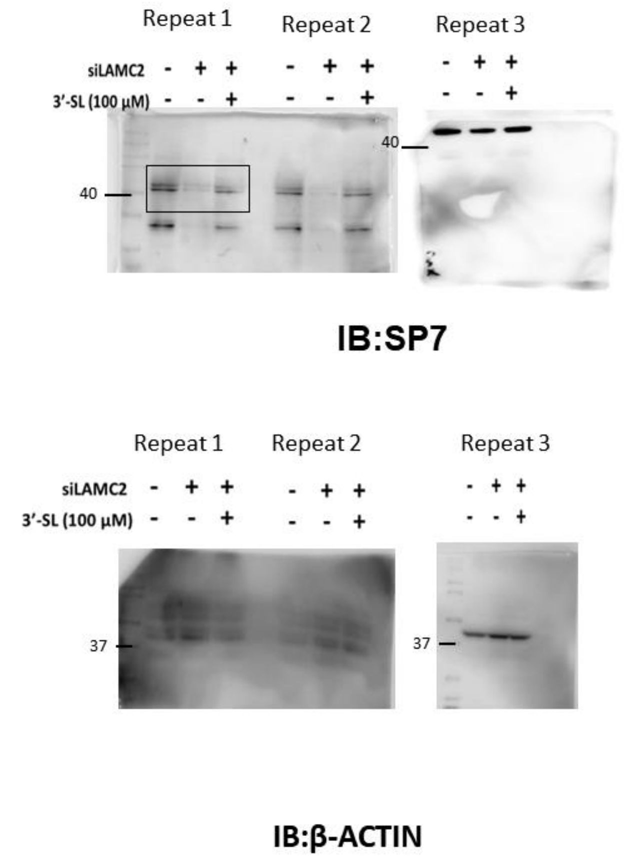

Figure 2 h

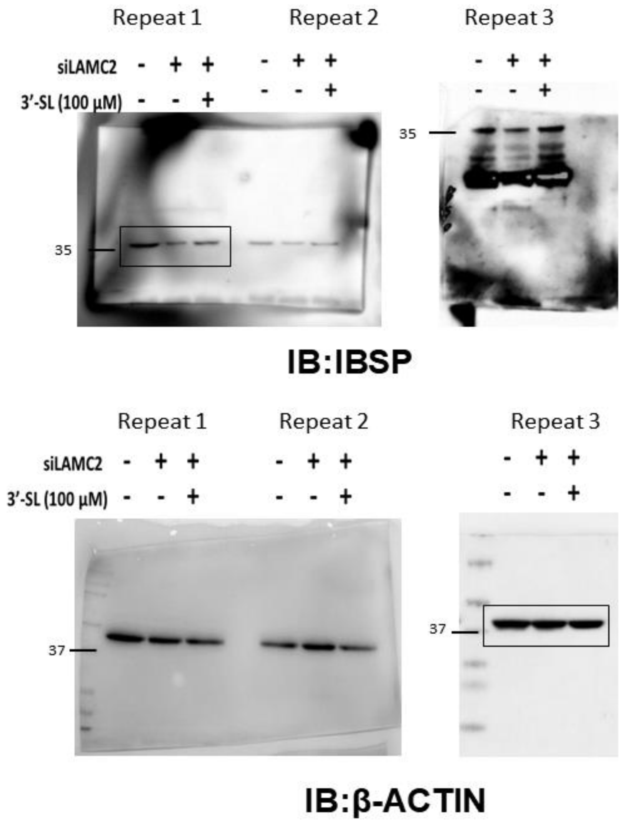

c

Figure 3 d

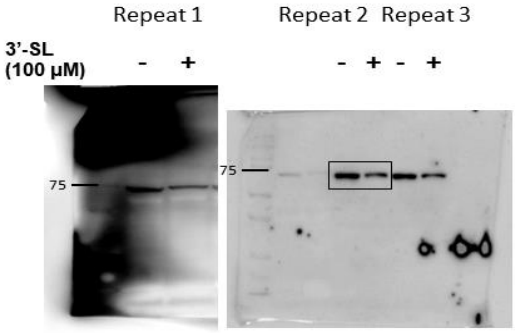

IB:PPARγ

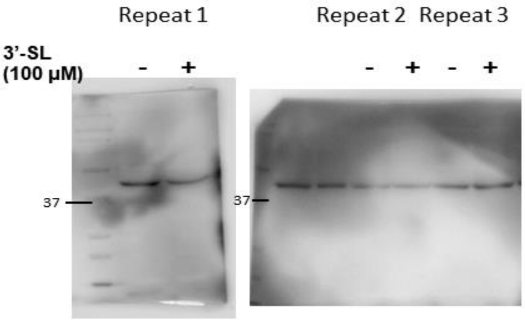

IB:β-ACTIN

Figure 3 d

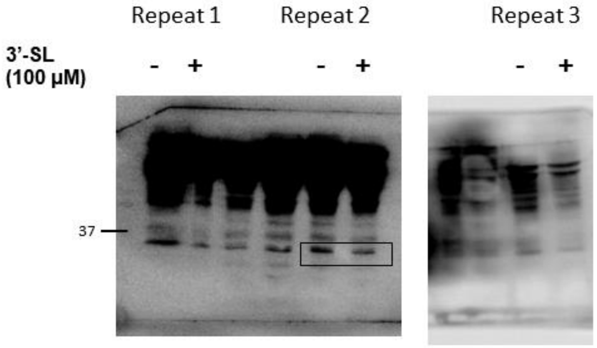

IB:C/EBPα

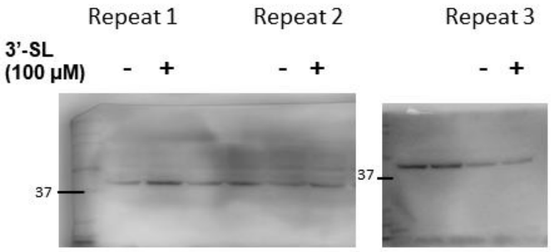

IB:β-ACTIN

Figure 3 d

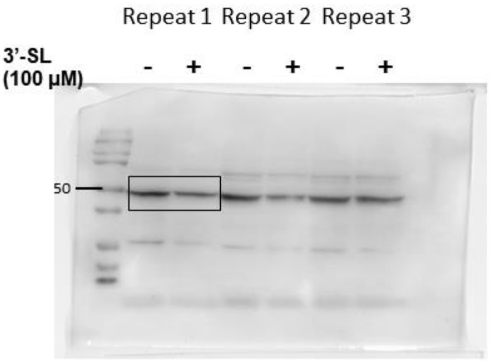

IB:C/EBPβ

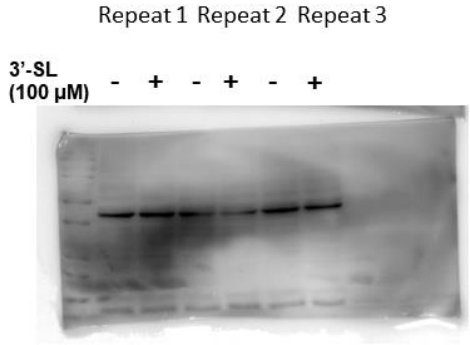

IB:β-ACTIN

Figure 3 d

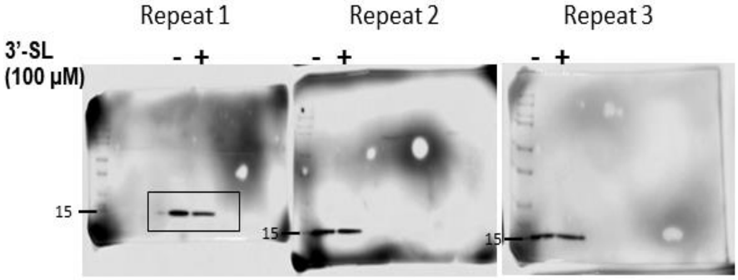

IB:FABP4

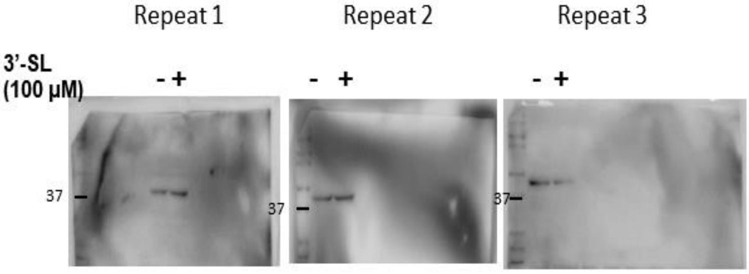

IB:β-ACTIN

Figure 3 d

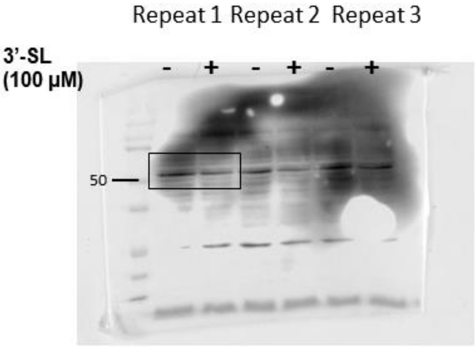

IB:PLIN1

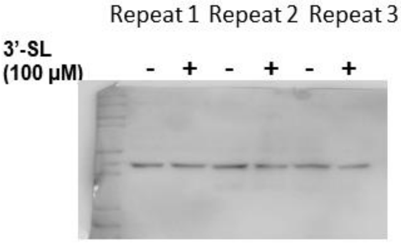

IB:β-ACTIN

Figure 3 d

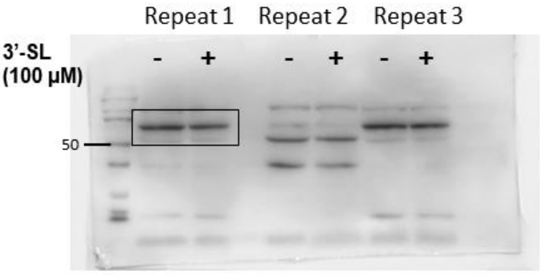

IB:LPL

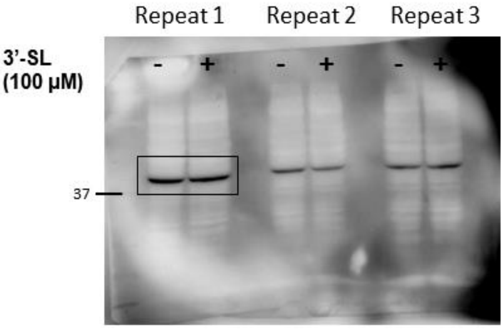

IB:β-ACTIN

Figure 3 d

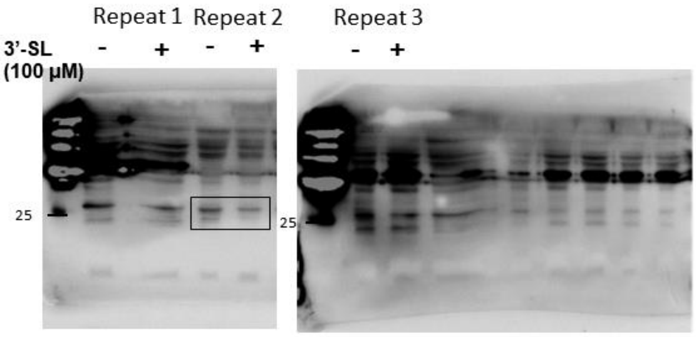

IB:ADIPOQ

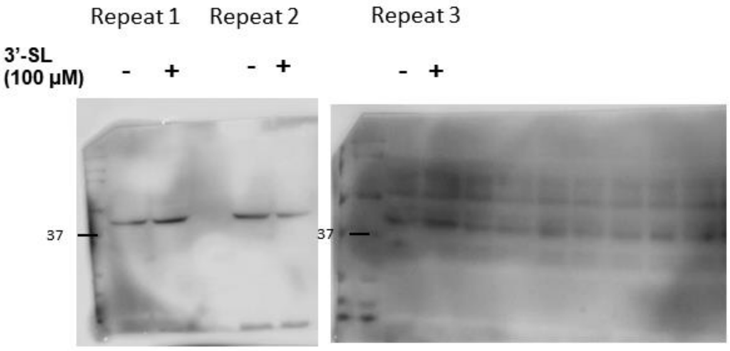

IB:β-ACTIN

d

Figure 4 d

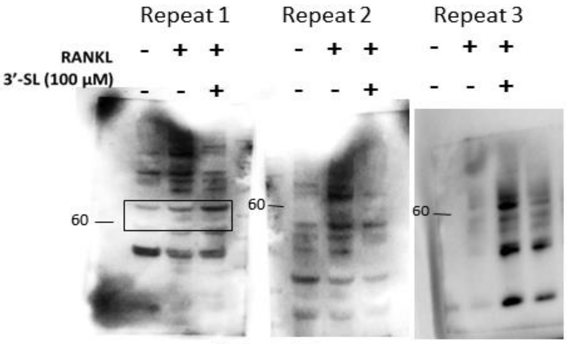

IB:c-Fos

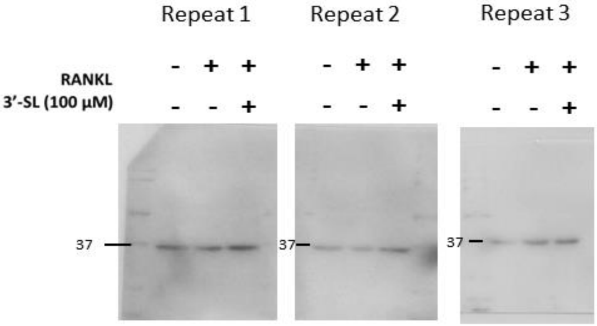

IB:GAPDH

Figure 4 d

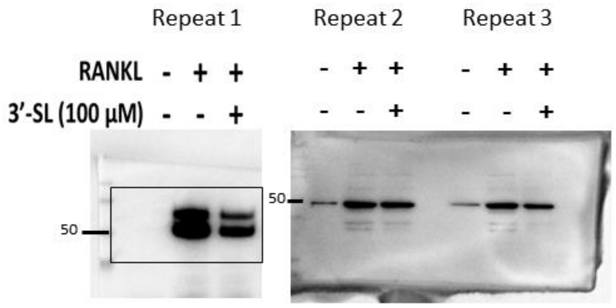

IB:MMP9

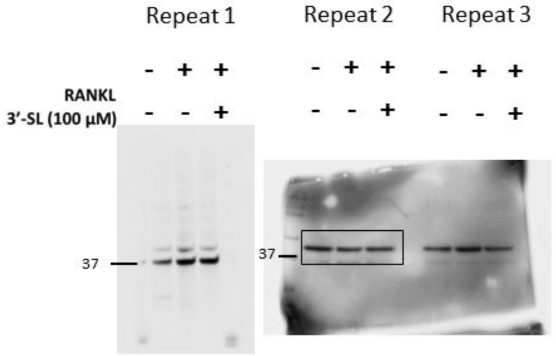

IB:GAPDH

Figure 4 d

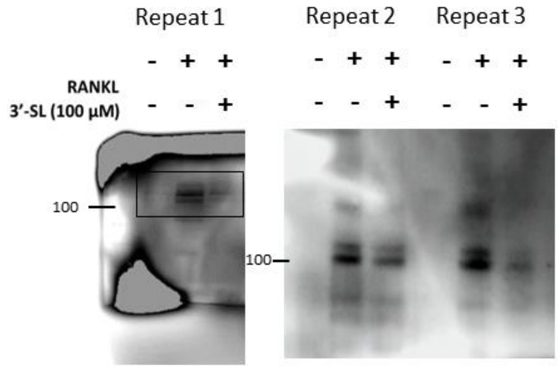

IB:NFATc1

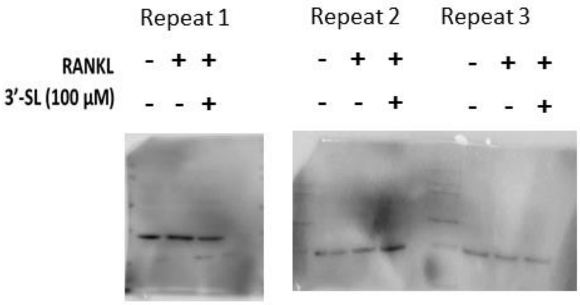

IB:GAPDH

Figure 4 h

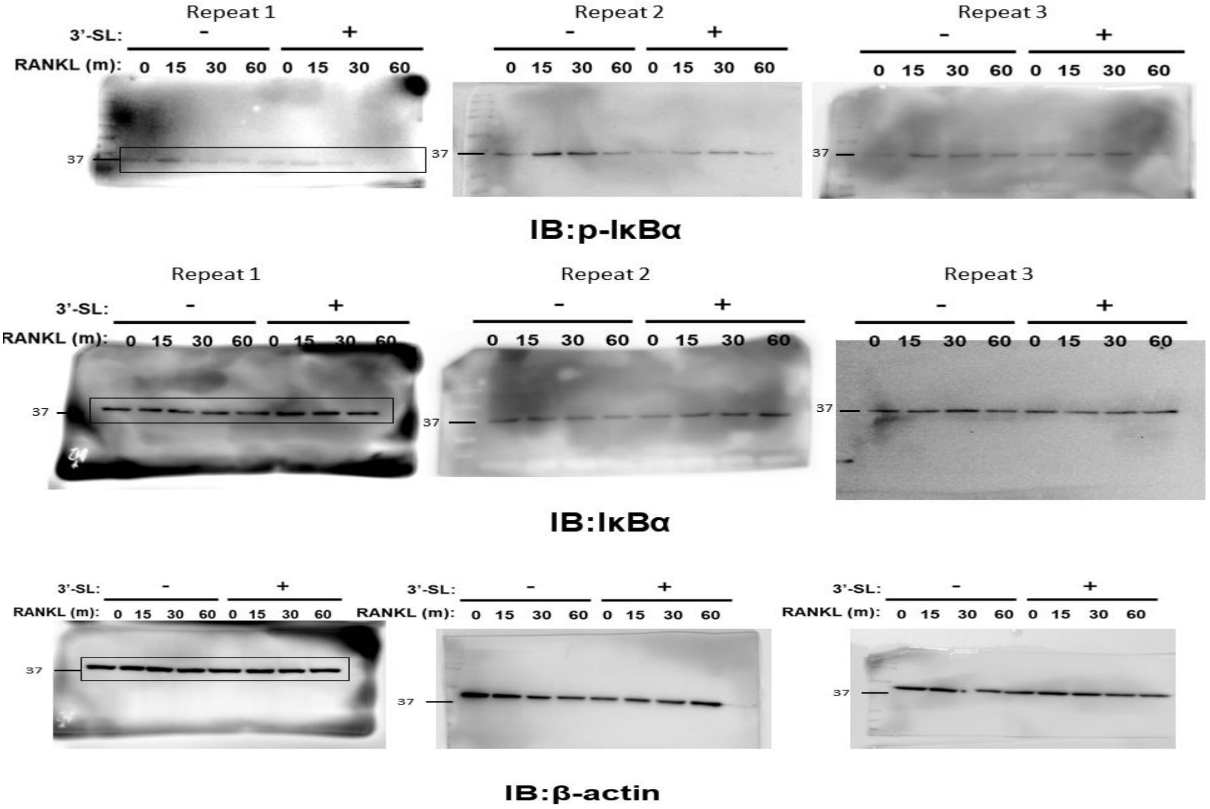

Figure 4 h

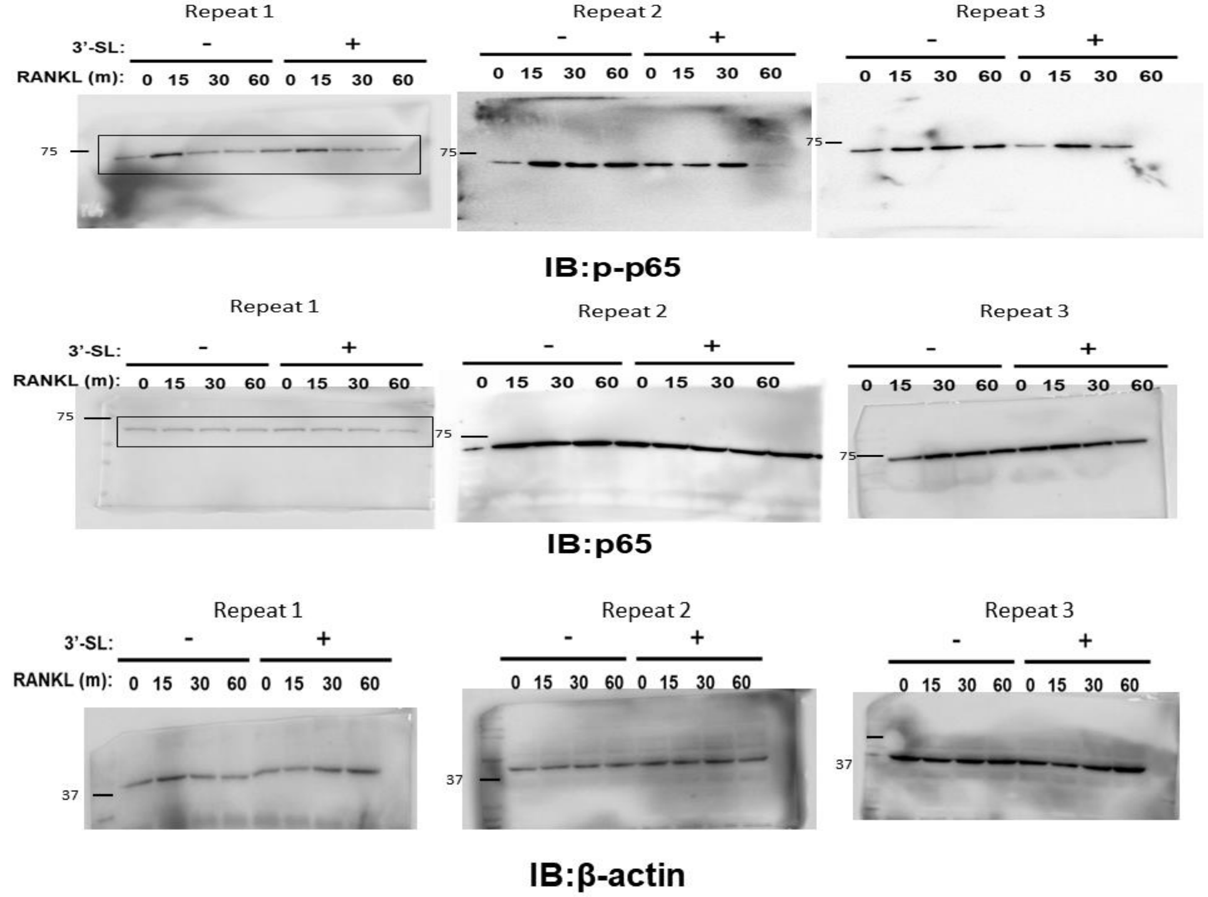

Figure 4 j

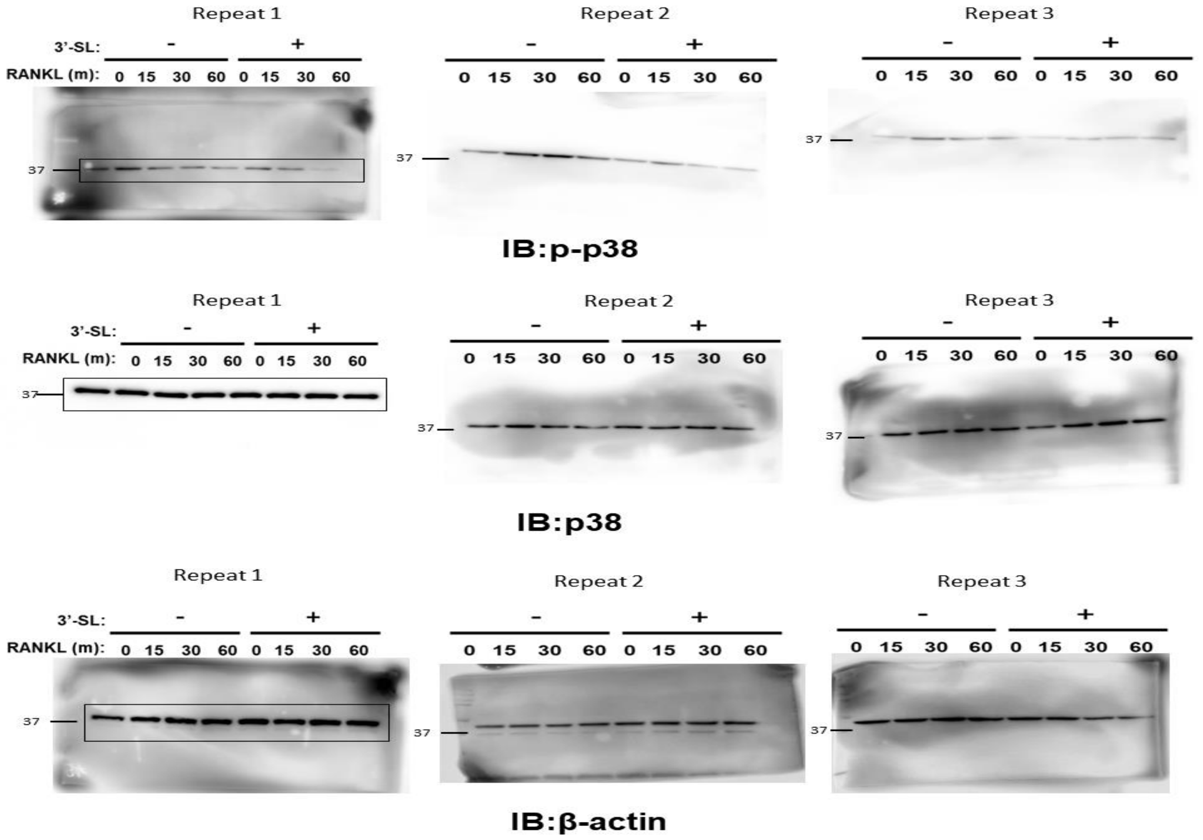

Figure 4 j

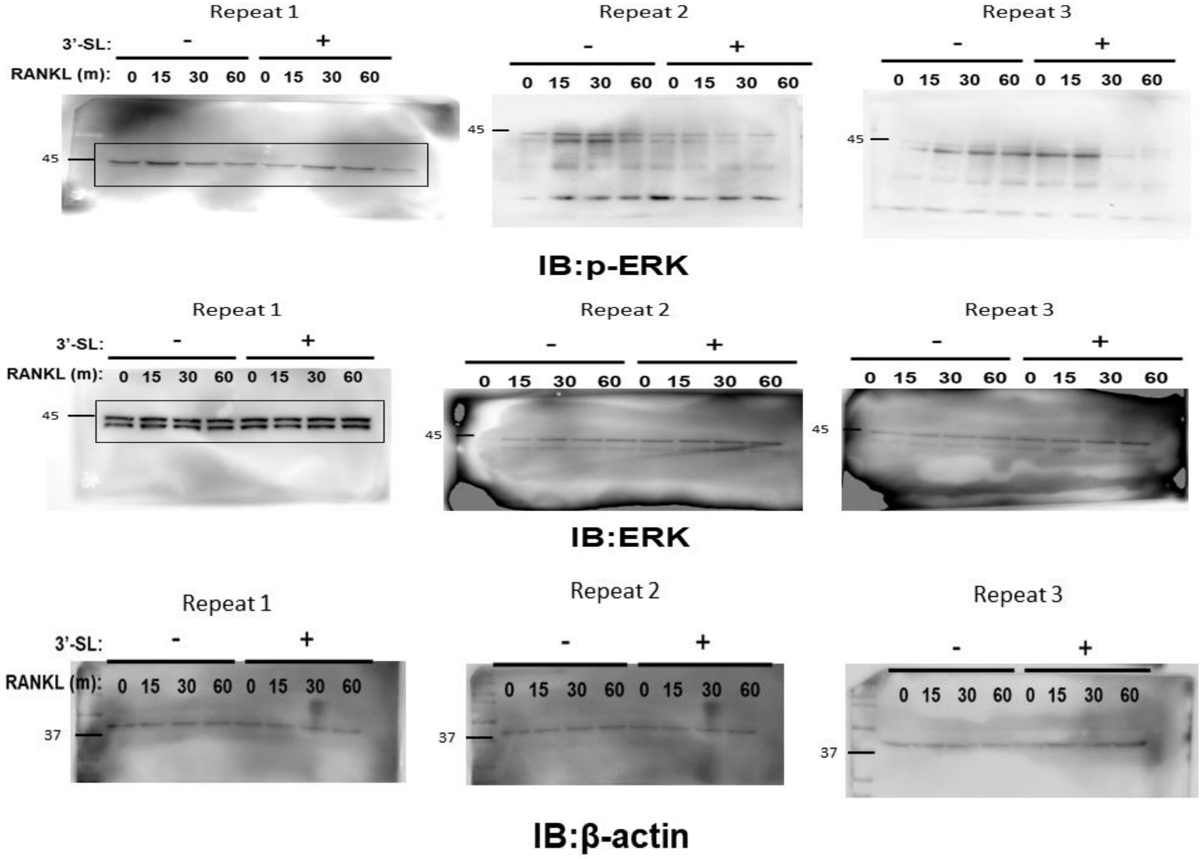

Figure 4 j

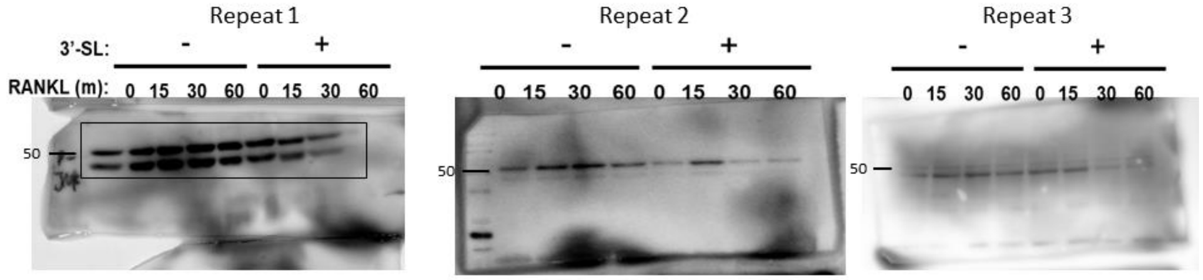

IB:p-JNK

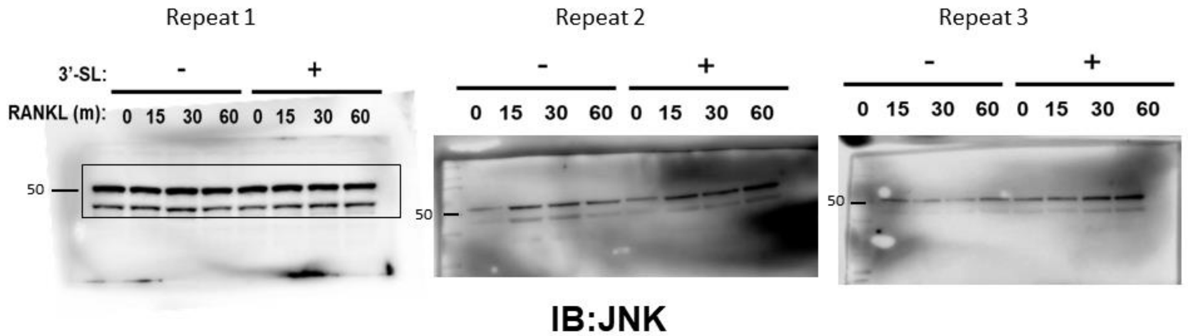

IB:JNK

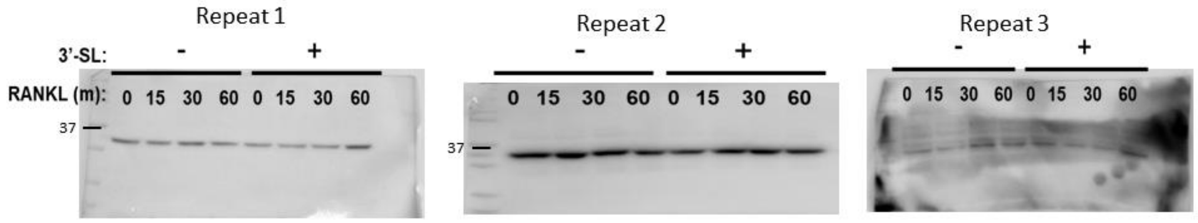

IB:β-actin

e

Figure 6 c

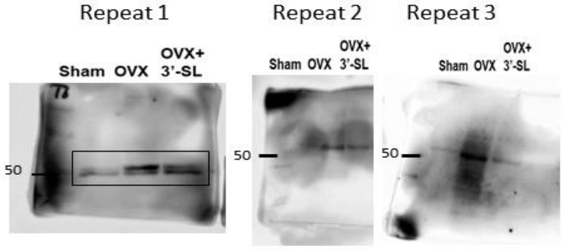

IB:Traf6

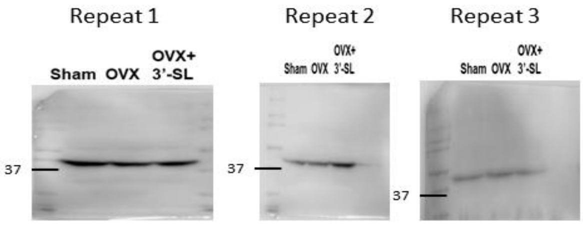

IB:β-actin

Figure 6 c

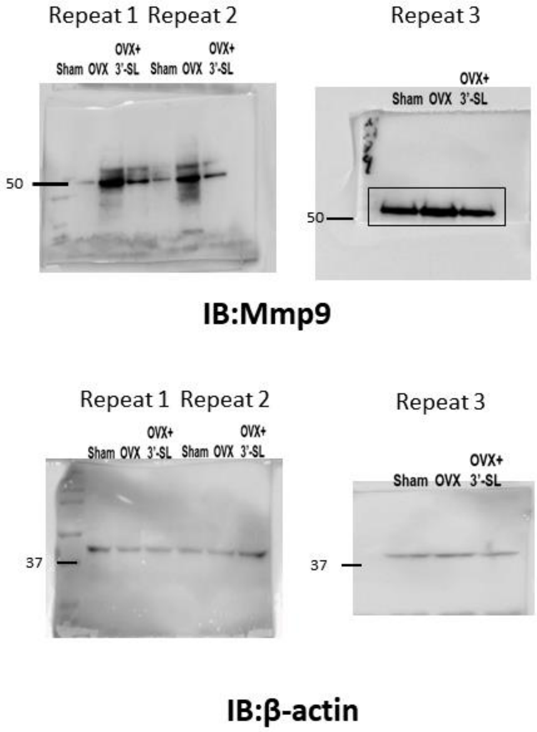

Figure 6 c

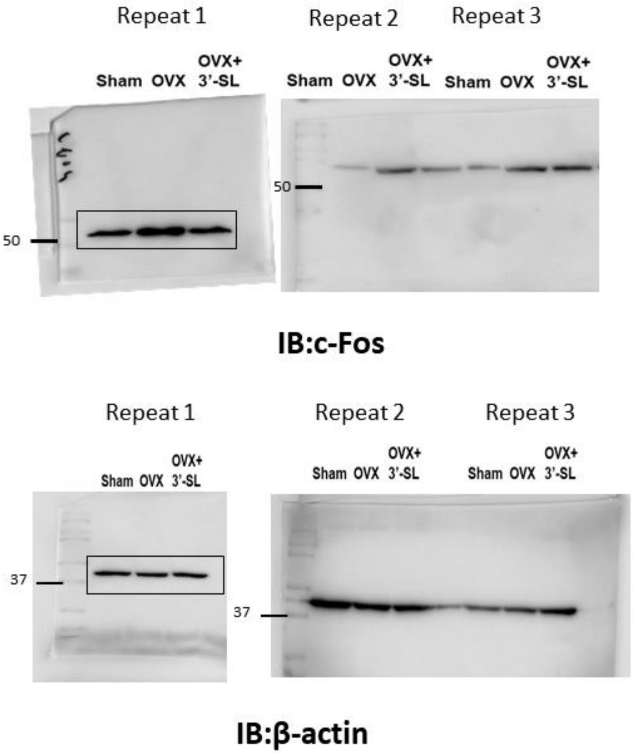

Figure 6 c

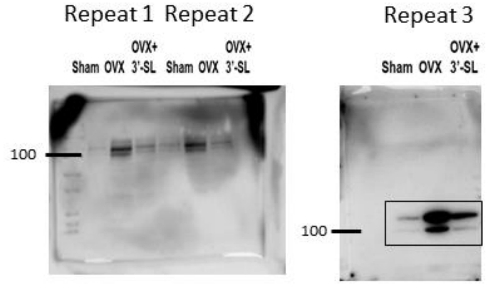

IB:Nfatc1

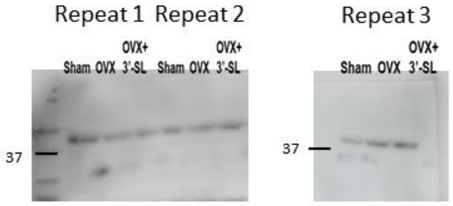

IB:β-actin

Figure 6 d

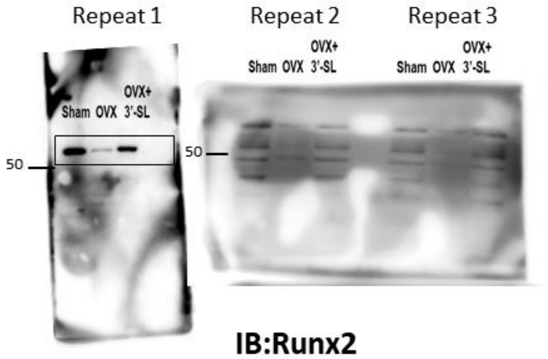

IB:Runx2

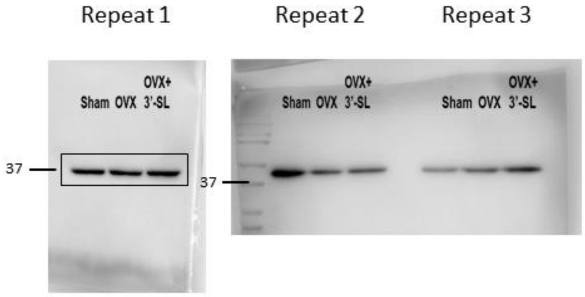

IB:β-actin

Figure 6 d

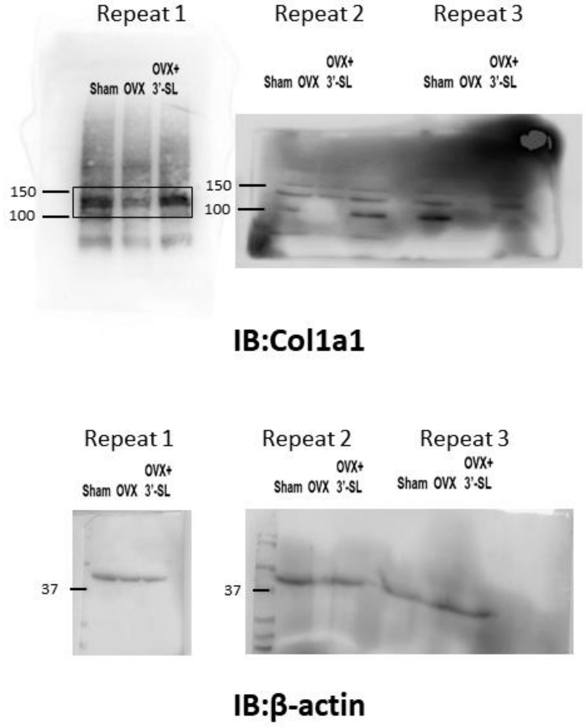

Figure 6 d

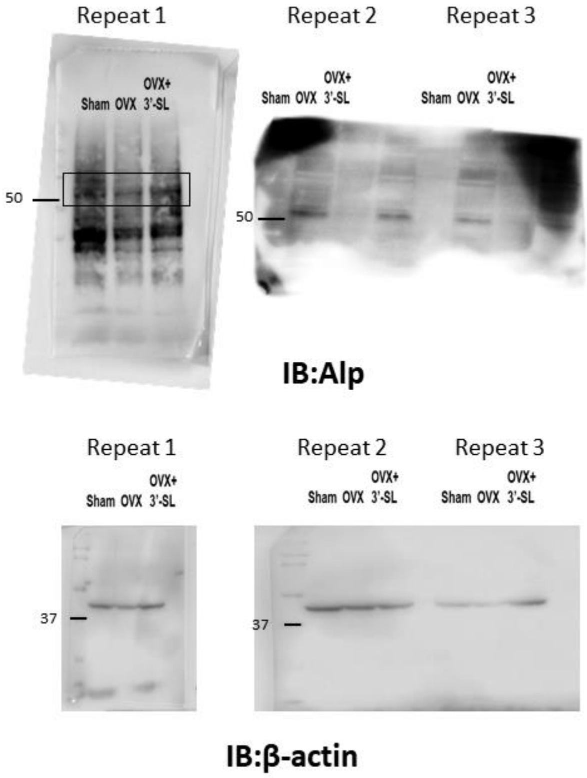

Supplementary Figure 3. Uncropped blots of western blots performed in this study.

a. Uncropped western blot images of Figure 1d. The bands in black boxes are presented in Figure 1d. GAPDH is used as a loading control.

b. Uncropped western blot images of Figure 2f and 2h. The bands in black boxes are presented in Figure 2f and 2h. GAPDH or β-ACTIN are used as a loading control.

c. Uncropped western blot images of Figure 3d. The bands in black boxes are presented in Figure 3d. β-ACTIN is used as a loading control.

d. Uncropped western blot images of Figure 4d, 4h, and 4j. A hole in the Figure 4d (NFATc1) is due to trapped air bubbles. The bands in black boxes are presented in Figure 4d, 4h, and 4j. GAPDH or β-ACTIN are used as a loading control.

e. Uncropped western blot images of Figure 6c and 6d. The bands in black boxes are presented in Figure 6c and 6d. β-ACTIN is used as a loading control.

Supplementary Table 1. Specific primer pairs used in reverse transcription-polymerase chain reaction analysis

Gene names		Sequence (5′ → 3′)
Human *ADIPOQ*	Forward	CAGGCCGTGATGGCAGAGATG
	Reverse	GGTTTCACCGATGTCTCCCTTAG
Human *ALP*	Forward	GCTGTAAGGACATCGCCTACCA
	Reverse	CCTGGCTTTCTCGTCACTCTCA
Human *C/EBPα*	Forward	AGGAGGATGAAGCCAAGCAGCT
	Reverse	AGTGCGCGATCTGGAACTGCAG
Human *C/EBPβ*	Forward	AGAAGACCGTGGACAAGCACAG
	Reverse	CTCCAGGACCTTGTGCTGCGT
Human *COL1A1*	Forward	GATTCCCTGGACCTAAAGGTGC
	Reverse	AGCCTCTCCATCTTTGCCAGCA
Human *FABP4*	Forward	ACGAGAGGATGATAAACTGGTGG
	Reverse	GCGAACTTCAGTCCAGGTCAAC
Human *GAPDH*	Forward	GTCTCCTCTGACTTCAACAGCG
	Reverse	ACCACCCTGTTGCTGTAGCCAA
Human *HPRT*	Forward	CATTATGCTGAGGATTTGGAAAGG
	Reverse	CTTGAGCACACAGAGGGCTACA
Human *IBSP*	Forward	GGCAGTAGTGACTCATCCGAAG
	Reverse	GAAAGTGTGGTATTCTCAGCCTC
Human *LAMC2*	Forward	TACAGAGCTGGAAGGCAGGATG
	Reverse	GTTCTCTTGGCTCCTCACCTTG
Human *LPL*	Forward	CTGCTGGCATTGCAGGAAGTCT
	Reverse	CATCAGGAGAAAGACGACTCGG
Human *OCN*	Forward	CGCTACCTGTATCAATGGCTGG
	Reverse	CTCCTGAAAGCCGATGTGGTCA
Human *OPN*	Forward	CGAGGTGATAGTGTGGTTTATGG
	Reverse	GCACCATTCAACTCCTCGCTTTC
Human *PLIN1*	Forward	GCGGAATTTGCTGCCAACACTC
	Reverse	AGACTTCTGGGCTTGCTGGTGT
Human *PPARγ*	Forward	AGCCTGCGAAAGCCTTTTGGTG
	Reverse	GGCTTCACATTCAGCAAACCTGG
Human *RUNX2*	Forward	CCCAGTATGAGAGTAGGTGTCC
	Reverse	GGGTAAGACTGGTCATAGGACC
Human *SP7*	Forward	TTCTGCGGCAAGAGGTTCACTC
	Reverse	GTGTTTGCTCAGGTGGTCGCTT
Mouse *Acp5*	Forward	GGGAAATGGCCAATGCCAAAGAGA
	Reverse	TCGCACAGAGGGATCCATGAAGTT
Mouse *Alp*	Forward	CCAGAAAGACACCTTGACTGTGG
	Reverse	TCTTGTCCGTGTCGCTCACCAT
Mouse *Col1a1*	Forward	CCTCAGGGTATTGCTGGACAAC
	Reverse	CAGAAGGACCTTGTTTGCCAGG
Mouse *Ctsk*	Forward	AGCAGAACGGAGGCATTGACTC
	Reverse	CCCTCTGCATTTAGCTGCCTTTG
Mouse *Dcstamp*	Forward	TCCTCCATGAACAAACAGTTCCAA
	Reverse	AGACGTGGTTTAGGAATGCAGCTC
Mouse *Fos*	Forward	GGAGAATCCGAAGGGAACGG
	Reverse	GCAATCTCAGTCTGCAACGC
Mouse *Hprt*	Forward	CTGGTGAAAAGGACCTCTCGAAG

	Reverse	CCAGTTTCACTAATGACACAAACG
Mouse *Nfatc1*	Forward	GGTAACTCTGTCTTTCTAACCTTAAGCTC
	Reverse	GTGATGACCCCAGCATGCACCAGTCACAG
Mouse *Ocstamp*	Forward	ATGAGGACCATCAGGGCAGCCACG
	Reverse	GGAGAAGCTGGGTCAGTAGTTCGT
Mouse *Runx2*	Forward	CCTGAACTCTGCACCAAGTCCT
	Reverse	TCATCTGGCTCAGATAGGAGGG

Supplementary Table 2. Details of antibodies used for western blotting

Protein	Source	Identifier	Dilution
AKT	Cell Signaling Technology	9272	1:1000
Adiponectin	ABclonal	A2543	1:1000
ALP	Abcam	ab65834	1:1000
β-Actin	Santa Cruz Biotechnology	sc-47778	1:1000
C/EBPα	ABclonal	A0904	1:1000
C/EBPβ	ABclonal	A0711	1:1000
c-Fos	Cell Signaling Technology	2250	1:1000
COL1A1	ABclonal	A1352	1:1000
ERK1/2	Cell Signaling Technology	4695	1:1000
FABP4	Cell Signaling Technology	50699	1:1000
GAPDH	Santa Cruz Biotechnology	sc-47724	1:1000
IBSP	Abcam	ab52128	1:1000
IκBα	Cell Signaling Technology	9242	1:1000
JNK	Cell Signaling Technology	9252	1:1000
LAMC2	Abcam	ab210959	1:1000
Lipoprotein lipase	ABclonal	A16252	1:1000
MMP9	Cell Signaling Technology	3852	1:1000
NFATc1	Thermo Fisher Scientific	MA3-024	1:1000
p38	Santa Cruz Biotechnology	sc-7972	1:1000
p65	Cell Signaling Technology	8242	1:1000
p-AKT	Cell Signaling Technology	9611	1:1000
Perilipin A	ABclonal	A16295	1:1000
p-ERK	Cell Signaling Technology	4370	1:1000
PI3K	Santa Cruz Biotechnology	sc-1637	1:1000
p-IκBα	Santa Cruz Biotechnology	sc-8404	1:1000
p-JNK	Cell Signaling Technology	4668	1:1000
p-p38	Cell Signaling Technology	4511	1:1000
p-p65	Cell Signaling Technology	3033	1:1000
PPARγ	Cell Signaling Technology	2443	1:1000
p-PI3K	Cell Signaling Technology	4228	1:1000
RUNX2	Cell Signaling Technology	12556	1:1000
SP7	Abcam	ab209484	1:1000
TRAF6	Santa Cruz Biotechnology	sc-8409	1:1000
