# Supplementary material for: 3′-Sialyllactose alleviates bone loss by regulating bone homeostasis
Source: Commun Biol. 2024 Jan 19;7:110. doi: 10.1038/s42003-024-05796-4 (PMC10798968; doi:10.1038/s42003-024-05796-4)
Supplement: Supplementary file 3 — Description of Additional Supplementary Files [file 42003_2024_5796_MOESM3_ESM.pdf]

Description of Additional Supplementary Files

File Name: Supplementary Data 1

Description: Source data underlying main figures.

File Name: Supplementary Data 2

Description: The source data behind the figure 2b-c.
