# Supplementary material for: 3′-Sialyllactose alleviates bone loss by regulating bone homeostasis
Source: Commun Biol. 2024 Jan 19;7:110. doi: 10.1038/s42003-024-05796-4 (PMC10798968; doi:10.1038/s42003-024-05796-4)
Supplement: Supplementary file 6 — Reporting Summary [file 42003_2024_5796_MOESM6_ESM.pdf]

nature portfolio

Corresponding author(s): Sung-Rae Cho and Sung Hoon Kim

Last updated by author(s): Dec 14, 2023

Reporting Summary

Nature Portfolio wishes to improve the reproducibility of the work that we publish. This form provides structure for consistency and transparency in reporting. For further information on Nature Portfolio policies, see our Editorial Policies and the Editorial Policy Checklist.

Statistics

For all statistical analyses, confirm that the following items are present in the figure legend, table legend, main text, or Methods section.

n/a	Confirmed	
☐	☒	The exact sample size (*n*) for each experimental group/condition, given as a discrete number and unit of measurement
☐	☒	A statement on whether measurements were taken from distinct samples or whether the same sample was measured repeatedly
☐	☒	The statistical test(s) used AND whether they are one- or two-sided<br>*Only common tests should be described solely by name; describe more complex techniques in the Methods section.*
☒	☐	A description of all covariates tested
☒	☐	A description of any assumptions or corrections, such as tests of normality and adjustment for multiple comparisons
☒	☐	A full description of the statistical parameters including central tendency (e.g. means) or other basic estimates (e.g. regression coefficient) AND variation (e.g. standard deviation) or associated estimates of uncertainty (e.g. confidence intervals)
☐	☒	For null hypothesis testing, the test statistic (e.g. *F*, *t*, *r*) with confidence intervals, effect sizes, degrees of freedom and *P* value noted<br>*Give P values as exact values whenever suitable.*
☒	☐	For Bayesian analysis, information on the choice of priors and Markov chain Monte Carlo settings
☒	☐	For hierarchical and complex designs, identification of the appropriate level for tests and full reporting of outcomes
☒	☐	Estimates of effect sizes (e.g. Cohen's *d*, Pearson's *r*), indicating how they were calculated

Our web collection on statistics for biologists contains articles on many of the points above.

Software and code

Policy information about availability of computer code

Data collection	No software was used to collect the data.
Data analysis	All data was statistically analyzed with SPSS Version 26.0 (IBM corporation), and all graph was expressed using Prism version 10.0 (Graphpad software).

For manuscripts utilizing custom algorithms or software that are central to the research but not yet described in published literature, software must be made available to editors and reviewers. We strongly encourage code deposition in a community repository (e.g. GitHub). See the Nature Portfolio guidelines for submitting code & software for further information.

Data

Policy information about availability of data

All manuscripts must include a data availability statement. This statement should provide the following information, where applicable:

- Accession codes, unique identifiers, or web links for publicly available datasets
- A description of any restrictions on data availability
- For clinical datasets or third party data, please ensure that the statement adheres to our policy

Sequencing data have been uploaded to the Sequence Read Archive under accession number BioProject: PRJNA930641

Research involving human participants, their data, or biological material

Policy information about studies with human participants or human data. See also policy information about sex, gender (identity/presentation), and sexual orientation and race, ethnicity and racism.

Reporting on sex and gender	N/A
Reporting on race, ethnicity, or other socially relevant groupings	N/A
Population characteristics	N/A
Recruitment	N/A
Ethics oversight	N/A

Note that full information on the approval of the study protocol must also be provided in the manuscript.

Field-specific reporting

Please select the one below that is the best fit for your research. If you are not sure, read the appropriate sections before making your selection.

☒ Life sciences ☐ Behavioural & social sciences ☐ Ecological, evolutionary & environmental sciences

For a reference copy of the document with all sections, see nature.com/documents/nr-reporting-summary-flat.pdf

Life sciences study design

All studies must disclose on these points even when the disclosure is negative.

Sample size	IN VITRO experiments: n=3, IN VIVO experiments: n=5-10.
Data exclusions	No data was excluded from analysis.
Replication	Replicate experiments were successful.
Randomization	All animals within a group were randomized and assigned each group.
Blinding	Blinding was not relevant to this study.

Reporting for specific materials, systems and methods

We require information from authors about some types of materials, experimental systems and methods used in many studies. Here, indicate whether each material, system or method listed is relevant to your study. If you are not sure if a list item applies to your research, read the appropriate section before selecting a response.

Materials & experimental systems

n/a	Involved in the study	
☐	☒	Antibodies
☐	☒	Eukaryotic cell lines
☒	☐	Palaeontology and archaeology
☐	☒	Animals and other organisms
☒	☐	Clinical data
☒	☐	Dual use research of concern
☒	☐	Plants

Methods

n/a	Involved in the study	
☒	☐	ChIP-seq
☒	☐	Flow cytometry
☒	☐	MRI-based neuroimaging

Antibodies

Antibodies used	Anti-AKT (Cell Signaling Technology, 9272), Anti-Adiponectin (ABclonal, A2543), Anti-ALP (Abcam,ab65834), Anti-β-Actin (Santa Cruz Biotechnology, sc-47778), Anti-C/EBPα (ABclonal, A0904), Anti-C/EBPβ (ABclonal, A0711), Anti-cFos (Cell Signaling Technology, 2250), Anti-COL1A1 (ABclonal, A1352), Anti-ERK1/2 (Cell Signaling Technology, 4695), Anti-FABP4 (Cell Signaling Technology, 50699), Anti-GAPDH (Santa Cruz Biotechnology, sc-47724), Anti-IBSP (Abcam, ab52128), Anti-IκBα (Cell Signaling Technology, 9242), Anti-JNK (Cell

	Signaling Technology, 9252), Anti-LAMC2 (Abcam, ab210959), Anti-Lipoprotein lipase (ABclonal, A16252), Anti-MMP9 (Cell Signaling Technology, 3852), Anti-NFATc1 (Thermo Fisher Scientific, MA3-024), Anti-p38 (Santa Cruz Biotechnology, sc-7972), Anti-P65 (Cell Signaling Technology, 8242), Anti-p-AKT (Cell Signaling Technology, 9611), Anti-Perilipin A (ABclonal, A16295), Anti-p-ERK (Cell Signaling Technology, 4370), Anti-PI3K (Santa Cruz Biotechnology, sc-1637), Anti-p-IκBα (Santa Cruz Biotechnology, sc-8404), Anti-p-JNK (Cell Signaling Technology, 4668), Anti-p-p38 (Cell Signaling Technology, 4511), Anti-p-P65 (Cell Signaling Technology, 3033), Anti-PPARγ (Cell Signaling Technology, 2443), Anti-p-PI3K (Cell Signaling Technology, 4228), Anti-RUNX2 (Cell Signaling Technology 12556), Anti-SP7 (Abcam, ab209484), Anti-TRAF6 (Santa Cruz Biotechnology, sc-8409).
Validation	All antibodies are commercially available and were commercially validated by the manufactures.

Eukaryotic cell lines

Policy information about cell lines and Sex and Gender in Research

Cell line source(s)	The hBMSCs and MC3T3-E1 (mouse pre- osteoblast) cells were purchased from the American Type Culture Collection (Manassas, VA, USA). The RAW 264.7 cells were obtained from Korean Cell Line Bank (Seoul, Korea).
Authentication	These cells were authenticated by the cell center, but not reauthenticated by our laboratory.
Mycoplasma contamination	These cells were tested by the cell center and confirmed that there was no mycoplasma contamination, but not retested by our laboratory.
Commonly misidentified lines (See ICLAC register)	None.

Animals and other research organisms

Policy information about studies involving animals; ARRIVE guidelines recommended for reporting animal research, and Sex and Gender in Research

Laboratory animals	The C57BL/6J mice
Wild animals	None
Reporting on sex	12 weeks old female
Field-collected samples	None
Ethics oversight	All the animal experiments and protocols were approved by the Committee on the Ethics of Animal Experiments of Yonsei University College of Medicine (permit number IACUC-2021-0167).

Note that full information on the approval of the study protocol must also be provided in the manuscript.

Plants

Seed stocks	N/A
Novel plant genotypes	N/A
Authentication	N/A
